# Enhancing a deep learning model for pulmonary nodule malignancy risk estimation in chest CT with uncertainty estimation

**DOI:** 10.1007/s00330-024-10714-7

**Published:** 2024-03-27

**Authors:** Dré Peeters, Natália Alves, Kiran V. Venkadesh, Renate Dinnessen, Zaigham Saghir, Ernst T. Scholten, Cornelia Schaefer-Prokop, Rozemarijn Vliegenthart, Mathias Prokop, Colin Jacobs

**Affiliations:** 1 Diagnostic Imaging Analysis Group, Medical Imaging Department, Radboud University Medical Center, Geert Grooteplein Zuid 10, 6525 GA Nijmegen, the Netherlands; 2 Department of Medicine, Section of Pulmonary Medicine, Herlev-Gentofte Hospital, Hellerup, Denmark; 3 Department of Clinical Medicine, University of Copenhagen, Copenhagen, Denmark; 4 Radiology Department, Meander Medical Center, Maatweg 3, 3813 TZ Amersfoort, The Netherlands; 5 Department of Radiology, University Medical Center Groningen, University of Groningen, Hanzeplein 1, 9700RB Groningen, The Netherlands

**Keywords:** Deep learning, Uncertainty, Tomography (X-ray computed), Multiple pulmonary nodules

## Abstract

**Objective:**

To investigate the effect of uncertainty estimation on the performance of a Deep Learning (DL) algorithm for estimating malignancy risk of pulmonary nodules.

**Methods and materials:**

In this retrospective study, we integrated an uncertainty estimation method into a previously developed DL algorithm for nodule malignancy risk estimation. Uncertainty thresholds were developed using CT data from the Danish Lung Cancer Screening Trial (DLCST), containing 883 nodules (65 malignant) collected between 2004 and 2010. We used thresholds on the 90th and 95th percentiles of the uncertainty score distribution to categorize nodules into certain and uncertain groups. External validation was performed on clinical CT data from a tertiary academic center containing 374 nodules (207 malignant) collected between 2004 and 2012. DL performance was measured using area under the ROC curve (AUC) for the full set of nodules, for the certain cases and for the uncertain cases. Additionally, nodule characteristics were compared to identify trends for inducing uncertainty.

**Results:**

The DL algorithm performed significantly worse in the uncertain group compared to the certain group of DLCST (AUC 0.62 (95% CI: 0.49, 0.76) vs 0.93 (95% CI: 0.88, 0.97); *p* < .001) and the clinical dataset (AUC 0.62 (95% CI: 0.50, 0.73) vs 0.90 (95% CI: 0.86, 0.94); *p* < .001). The uncertain group included larger benign nodules as well as more part-solid and non-solid nodules than the certain group.

**Conclusion:**

The integrated uncertainty estimation showed excellent performance for identifying uncertain cases in which the DL-based nodule malignancy risk estimation algorithm had significantly worse performance.

**Clinical relevance statement:**

Deep Learning algorithms often lack the ability to gauge and communicate uncertainty. For safe clinical implementation, uncertainty estimation is of pivotal importance to identify cases where the deep learning algorithm harbors doubt in its prediction.

**Key Points:**

• *Deep learning (DL) algorithms often lack uncertainty estimation, which potentially reduce the risk of errors and improve safety during clinical adoption of the DL algorithm.*

• *Uncertainty estimation identifies pulmonary nodules in which the discriminative performance of the DL algorithm is significantly worse.*

• *Uncertainty estimation can further enhance the benefits of the DL algorithm and improve its safety and trustworthiness.*

**Supplementary Information:**

The online version contains supplementary material available at 10.1007/s00330-024-10714-7.

## Introduction

The National Lung Screening Trial (NLST) and Dutch-Belgian NELSON lung cancer screening trial provide evidence that lung cancer mortality can be reduced by repeated screening of high-risk individuals with low-dose chest CT [1, 2]. This decrease is primarily attributed to early detection of lung cancer in stages I and II, where a favorable prognosis is more probable than in stages III or IV [3]. Lung cancer screening could therefore play a crucial role in reducing lung cancer mortality. However, a global shortage of radiologists can still lead to a delayed diagnosis [4].

Deep learning (DL) can be a helpful tool to contribute to reducing radiologists’ workload and assist radiologists in lung cancer diagnosis. Venkadesh et al proposed a DL algorithm for pulmonary nodule malignancy risk estimation that was internally and externally validated on data from the NLST and Danish Lung Cancer Screening Trial (DLCST), respectively [5, 6]. The algorithm showed excellent performance on par with thoracic radiologists for pulmonary nodule malignancy risk estimation in an external validation set [6].

An imperative aspect of the safe clinical implementation of such DL algorithms is the ability to gauge and communicate uncertainty [7]. Estimating uncertainty is crucial to identify situations where the algorithm harbors doubt about its predictions and helps identify cases prone to potential DL interpretation errors [8]. Leveraging this uncertainty estimation could optimize the clinical workflow by preventing the algorithm from making a diagnosis in cases of high uncertainty and referring these to clinical experts for further evaluation. Conversely, clinical experts may have more confidence in the algorithm’s prediction when certainty is high. This proactive integration of uncertainty estimates holds promise to minimize error risks, thereby elevating the safety profile of a clinically adopted algorithm [9]. Existing studies corroborate the efficacy of uncertainty estimation in filtering out ambiguous cases, thereby enhancing algorithm accuracy in the remaining cases [10–16]. However, while most studies primarily focus on refining algorithm accuracy and not on evaluating causes of uncertainty, a prevailing limitation lies in the inadequate identification of underlying trends responsible for inducing uncertainty. Addressing this limitation is important because without understanding what causes uncertainty, it is challenging to improve the reliability of a DL algorithm.

In this study, we build upon a previously developed DL algorithm and integrate a method for uncertainty estimation. Our aim was to investigate the performance of the algorithm for pulmonary nodule malignancy risk estimation when different uncertainty thresholds are applied and identify nodule characteristics responsible for inducing uncertainty in the algorithm.

## Methods

### Data collection

This retrospective study was conducted using data from two data sources: the Danish lung cancer screening trial (DLCST) [5] and a Dutch tertiary academic center (clinical dataset). DLCST was used as a development set for the uncertainty estimation method. The DLCST dataset consists of anonymized low-dose chest CT examinations that were acquired from participants in the screening trial between 2004 and 2010. Approval for DLCST was obtained from the Ethics Committee of Copenhagen County, and all participants provided informed consent. Two thoracic radiologists performed all nodule annotations in DLCST. The malignant status of nodules was confirmed by histological analysis, and for benign status, stability over at least 2 years of CT follow-up was used. For participants diagnosed with lung cancer, we included the first image on which the malignant nodule was annotated. For participants without a lung cancer diagnosis, we included nodule annotations from the baseline image.

**Fig. 1:**
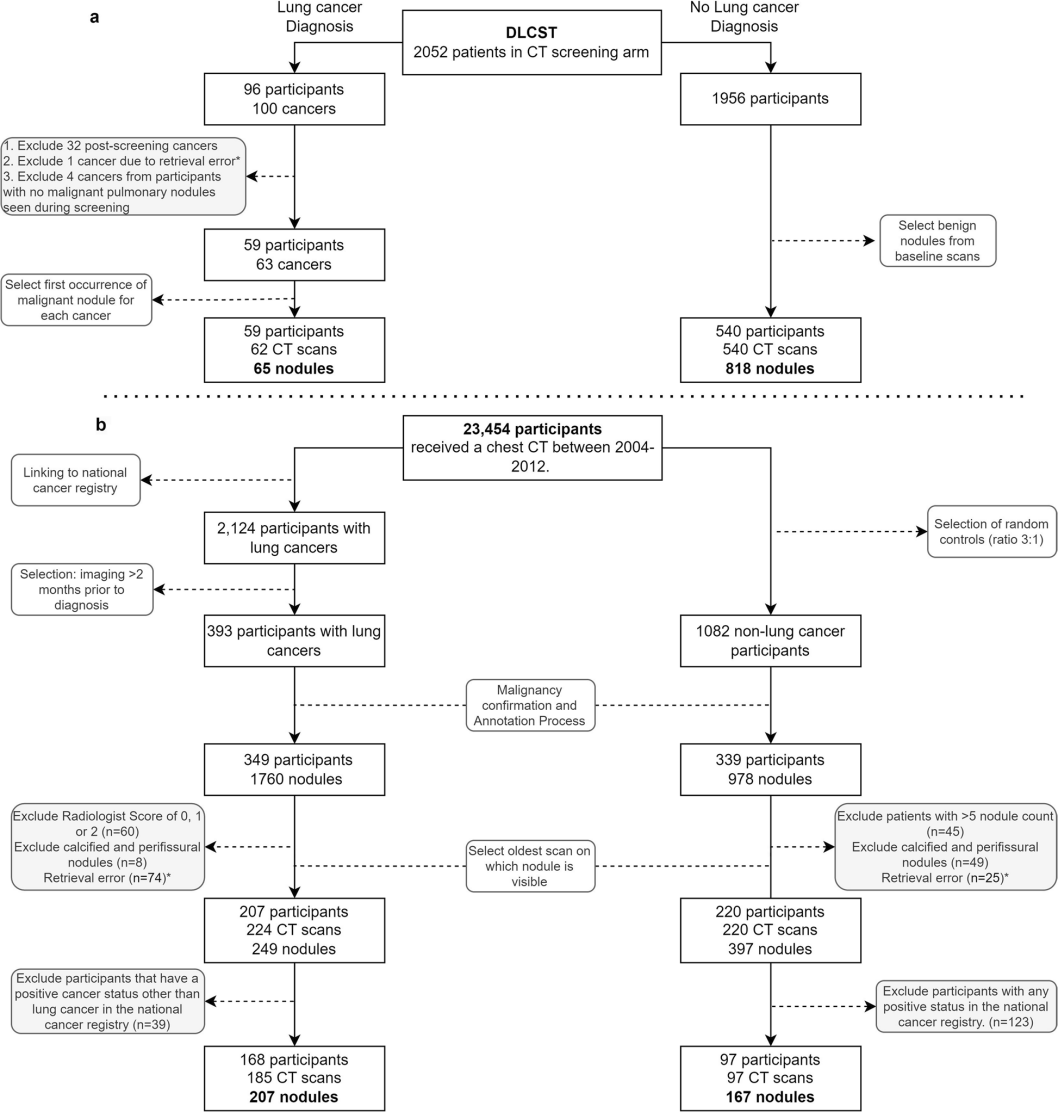
Flow chart of the data collection and selection of pulmonary nodules. **a** Nodules from the DLCST were used for the development of the uncertainty estimations. **b** Incidental nodules from the clinical dataset for validation of the uncertainty estimations. Retrieval errors may be due to anonymization, image quality, protected patients, or scan availability. Radiologist score of 0, 1, or 2 indicates no lesion, a benign nodule, or indeterminate nodule in the tumor-bearing lobe, respectively

The clinical dataset served as an external validation set, which consists of chest CT examinations in individuals aged 40 years and older acquired for clinical indications, with diverse CT scan protocols (with/without iodine contrast) between 2004 and 2012. The institutional review board waived the need for informed consent because of the retrospective design and data pseudonymization. Subjects were linked to the Dutch national cancer registry to gather information on lung cancer diagnosis until the end of 2014. Further details of the collection of the dataset are described in Chung et al (*Thorax*, 2018) [17]. We adjusted the set of malignant and benign nodules from Chung et al. by excluding all nodules from patients with a history of other primary cancers. We added this exclusion criterion to remove any participants with any potential metastases. For the clinical dataset, the malignant status of nodules was also confirmed by histological analysis, and for benign status, stability with a median of 5+ years of CT follow-up was used. A flow chart of the data collection and selection is shown in Figure 1 for both datasets.

### Uncertainty estimation

To gauge algorithm uncertainty, we first calculated the malignancy risk estimation score for all nodules in DLCST and the clinical dataset using a previously established DL-based ensemble algorithm [6]. This DL algorithm predicts a lung nodule malignancy risk score using an ensemble of 10 two-dimensional and 10 three-dimensional convolutional neural networks. The DL algorithm was trained and internally validated on data from NLST. Further details of the training procedure are described in Venkadesh et al (*Radiology*, 2020) [6]. Data from DLCST and the clinical dataset were not part of the training data of the DL algorithm.

To assess the ensemble’s variability and quantify uncertainty, the entropy was calculated for all 20 distinct DL outputs in the ensemble. Entropy provides a quantitative measure of the randomness of the DL outputs that can be used as an estimation of uncertainty for the predicted malignancy risk. Entropy has already shown its benefits in DL for medical image segmentation and grading of breast carcinoma in previous research [18, 19]. High values of entropy indicate that the DL algorithm is equally torn between predicting a benign or malignant outcome, whereas low entropy indicates that the DL algorithm is confident in its prediction. Entropy can therefore serve as an indicator of algorithm uncertainty. For instance, the entropy reaches its peak when all 20 networks make predictions around 0.5, suggesting uncertainty in classifying the malignancy risk of a nodule. In contrast, the entropy reaches its minimum when all predictions are clustered around either 0 or 1. The final uncertainty score was derived by averaging the entropy values from all 20 distinct DL outputs using the formula below:

$$\overline{H }=\frac{1}{N}{\sum }_{i=1}^{N}\left[-\left({p}_{i}{{\text{log}}}_{2}\left({p}_{i}\right)+\left(1-{p}_{i}\right){{\text{log}}}_{2}\left(1-{p}_{i}\right)\right)\right]$$

where $\overline{H }$ is the mean entropy, $N$ is the number of distinct DL outputs, and ${p}_{i}$ is the predicted malignancy risk for the positive class.

### Development set

**Fig. 2:**
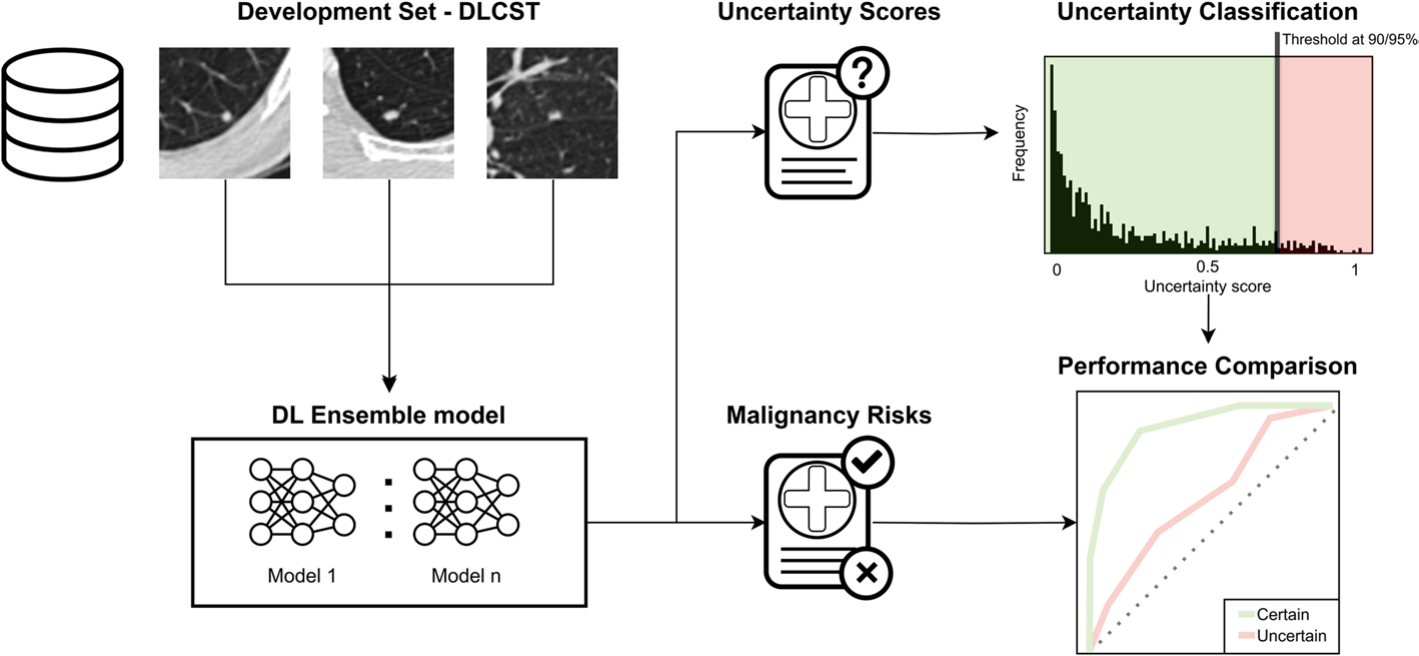
Schematic overview of how the uncertainty score is utilized to split the dataset into a certain and uncertain group. A nodule block (50x50x50mm) is used as input of the DL algorithm that outputs a malignancy risk and uncertainty score. The uncertainty score is determined based on the score of the individual algorithms in the ensemble. A 90th/95th percentile cut-off value on the uncertainty distribution of all nodules in the dataset is used to split it into a certain and uncertain group to compare algorithm performance. The uncertainty distribution is based on the mean entropy of the individual outputs of the DL algorithm

An uncertainty score, using mean entropy, was computed for each nodule in DLCST, resulting in a distribution of uncertainty scores across the entire dataset. Two cut-off values were determined at the 90th and 95th percentiles of this distribution, which were used as discriminative thresholds for classifying nodules into two distinct groups: certain and uncertain. These thresholds resulted in 90% and 95% of the data being retained as certain. This allows us to maintain the largest part of our data in a certain group, hereby mimicking a real-world scenario in which referring a high percentage of cases to human readers would limit and counteract the use of a DL algorithm. Additionally, this allows us to maintain enough data to assess the discriminative performance of the DL algorithm in the uncertain group. A schematic overview of this process is shown in Figure 2.

The discriminative performance of the DL algorithm for a nodule malignancy risk estimation task was compared between the full dataset and certain and uncertain groups using the 90th and 95th percentile thresholds. The discriminative performance of the DL algorithm was assessed by using the area under the receiver operating characteristics curve (AUC) and in terms of sensitivity at 95% specificity. By setting the specificity at 95%, we aimed to minimize false positives. Additionally, a subgroup analysis was conducted on nodule types (solid, part-solid, and non-solid) and nodule size. For nodule size subgroup analysis, nodules were categorized as small (<6mm), medium (≥ 6 to < 8mm), or large (≥8mm), following the size criteria from Lung-RADS 2022 [20]. The subgroup analysis was performed using the 90^th^ percentile threshold to make sure enough nodules remained in each subgroup of the uncertain cases for further analysis. This subgroup examination allows us to gain insights into the DL algorithm’s level of uncertainty within distinct subgroups and unveil nodule characteristics associated with uncertainty.

### External validation set

The uncertainty thresholds obtained from the DLCST dataset were applied to nodules of the clinical dataset to investigate whether the DL algorithm exhibits increased uncertainty when dealing with non-screening data. The 90th and 95th percentile thresholds, initially determined on DLCST, were externally validated on the incidentally detected nodules from the clinical dataset. These discriminative thresholds were again used to categorize the nodules into certain and uncertain groups. The performance of the DL algorithm for estimating pulmonary nodule malignancy risk was compared between the full set of nodules in the clinical dataset and certain and uncertain groups using 90th and 95th percentile thresholds of mean entropy.

Furthermore, a subgroup analysis was conducted to discern potential nodule characteristics associated with uncertainty using the 90^th^ percentile threshold of DLCST. This analysis employs the same nodule type and size categories as used for subgroup analysis on the DLCST data. Simultaneously, this approach enabled us to facilitate a comparison between the two datasets, bolstering our understanding of the algorithm's behavior and performance across nodules from distinct study populations.

### Statistical analysis

The independent samples t-test was conducted to establish statistical differences in nodule size of certain and uncertain benign and malignant nodules and in nodule size between DLCST and the clinical dataset. The chi-square test was conducted to establish statistical differences in nodule type and size (small, medium, large) of the full dataset and the certain and uncertain groups. For the Chi-square test, part-solid and non-solid types were grouped together as subsolids to account for their small sample size. DeLong’s test was conducted to assess statistical differences between the AUC of the full data and certain and uncertain groups. *p* values below 0.05 indicated a statistically significant difference.

## Results

### Participant and nodule characteristics

The DLCST dataset included 599 participants (mean age, 58 years ± 5 [SD]; 484 men) with 883 nodules in 602 CT examinations, and the clinical dataset included 265 participants (mean age, 64 years ± 10 [SD]; 233 men) with 374 nodules in 282 CT examinations. In participants with multiple CT examinations, the first detection of the individual nodules was not observed in the same CT examination. Table 1 shows the patient and nodule characteristics for both datasets. The benign nodules in DLCST were, on average, smaller than the benign nodules in the clinical dataset (*p* < 0.001). In DLCST and the clinical dataset, 85% and 81% of all benign nodules were solid, respectively (*p* = 0.305). The malignant nodules in DLCST were, on average, smaller than the malignant nodules in the clinical dataset (*p* < 0.001). In DLCST, 69% of all malignant nodules were solid, whereas in the clinical dataset, 90% of all malignant nodules were solid (p < 0.001).

**Table 1:** Patient and nodule characteristics for DLCST and the clinical dataset

	**DLCST**		**Clinical dataset**	
	**Benign**	**Malignant**	**Benign**	**Malignant**
	**(818)**	**(65)**	**(167)**	**(207)**
Mean age in years	57.9 ± 4.7	61.8 ± 4.8	63.8 ± 11.4	64.6 ± 9.0
Sex				
**Male**	446 (55)	38 (58)	111 (66)	122 (59)
**Female**	372 (45)	27 (42)	56 (34)	85 (41)
Nodule Type				
**Solid**	695 (85)	45 (69)	136 (81) †	187 (90) †
**Part-solid**	27 (3)	12 (19)	8 (5) †	15 (7) †
**Non-solid**	96 (12)	8 (12)	23 (14) †	5 (3) †
Nodule Size (mm)				
**Mean**	6.5 ± 5.1	14.6 ± 12.1	9.3 ± 7.2*	28.0 ± 17.9*
**Median**	5.0 [4.0, 7.0]	11.0 [8.0, 17.0]	6.8 [5.0, 10.9]	22.4 [15.4, 37.2]
Nodule Size				
**Small (<6mm)**	462 (56)	4 (6)	69 (41)*	0 (0)*
**Medium (6-8mm)**	169 (21)	8 (12)	29 (18)*	5 (2)*
**Large (>8mm)**	187 (23)	53 (82)	69 (41)*	202 (98)*

Note. Data are numbers of nodules, with percentages in parentheses, except where otherwise indicated. When median numbers are reported, brackets indicate the inter-quartile range from the 25th and 75th percentile. When mean data are reported, ± indicates standard deviation. *: significant difference between benign/malignant nodules of DLCST and the clinical dataset (*p*<.05). †: significant difference between benign/malignant solid and subsolid nodules in DLCST and the clinical dataset (*p*<.05)

### Development set

**Fig. 3:**
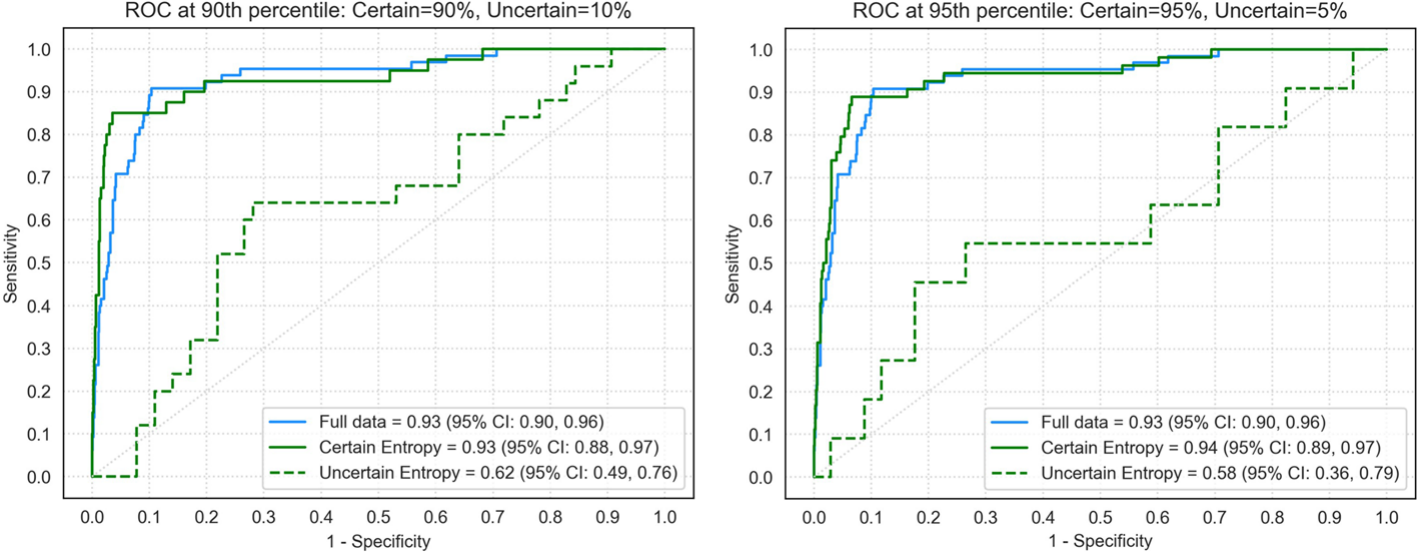
AUC for a nodule malignancy risk estimation task when using mean entropy to determine certain and uncertain cases of the DLCST dataset. AUC: area under the receiver operating curve

Uncertainty estimation scores were calculated across all 20 predictions of the ensemble algorithm based on mean entropy for all 883 nodules in the DLCST dataset. The entropy-based uncertainty score was 0.51 and 0.59 for the 90th and 95th percentile threshold, respectively. The algorithm achieved an AUC of 0.93 (95% CI: 0.90, 0.96) on the full dataset (Figure 3 and Table 2). With the 90th percentile threshold, the algorithm achieved an AUC of 0.93 (95% CI: 0.88, 0.97) for the certain group and an AUC of 0.62 (95% CI: 0.49, 0.76) for the uncertain group. With the 95th percentile threshold, the algorithm achieved an AUC of 0.94 (95% CI: 0.89, 0.97) for the certain group and an AUC of 0.58 (95% CI: 0.36, 0.79) for the uncertain group. DeLong’s test showed a significant difference in the performance of the DL algorithm between the certain group and the uncertain group (*p* < 0.001 and *p* = 0.003) for the 90th and 95th percentile, respectively. No significant differences were found between the full dataset and the certain group for the 90th and 95th percentile (*p* = 0.935 and *p* = 0.832, respectively). At 95% specificity, the DL algorithm achieved a sensitivity of 68% on the full dataset, while a sensitivity of 82% was achieved on the certain group of the 90th percentile.

**Table 2:** Performance of the DL algorithm on the full DLCST and the clinical dataset and across all uncertainty thresholds. Sensitivity was computed at a specificity of 95%

		**DLCST**			**Clinical dataset**		
		**AUC**	***p* value**	**Sensitivity**	**AUC**	***p* value**	**Sensitivity**
Full data		0.93 (0.90, 0.96)		68 (56, 82)	0.88 (0.84, 0.91)		32 (17, 53)
90th percentile							
certain		0.93 (0.88, 0.97)	0.935	83 (71, 95)	0.90 (0.86, 0.94)	0.342	34 (17, 54)
uncertain		0.62 (0.49, 0.76)	<0.001	4 (0, 47)	0.62 (0.50, 0.73)	<0.001	20 (0, 45)
95th percentile							
certain		0.94 (0.89, 0.97)	0.832	75 (62, 90)	0.89 (0.86, 0.93)	0.566	35 (17, 55)
	uncertain	0.58 (0.36, 0.79)	0.003	15 (0, 67)	0.64 (0.49, 0.78)	0.004	16 (0, 40)

Note. Parentheses indicate the 95% Confidence Interval calculated using bootstrapping (*n*=1000)

### External validation set

By applying the 90th percentile DLCST uncertainty threshold, 27% of the clinical dataset was determined to be uncertain in comparison with 10% of the DLCST dataset. With the 95th percentile DLCST uncertainty threshold, 15% of the clinical dataset was determined to be uncertain in comparison with the 5% on the DLCST dataset.

**Fig. 4:**
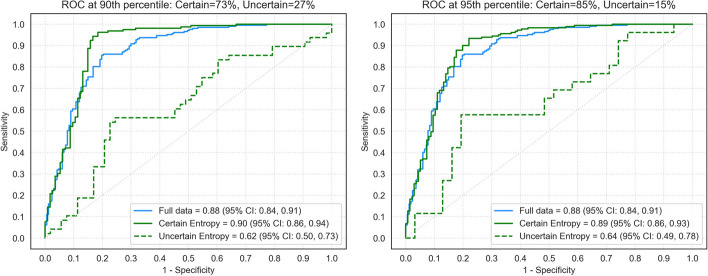
AUC for a nodule malignancy risk estimation task when using the DLCST 90th and 95th percentile threshold of mean entropy to determine certain and uncertain cases of the clinical dataset. AUC: area under the receiver operating curve

**Fig. 5:**
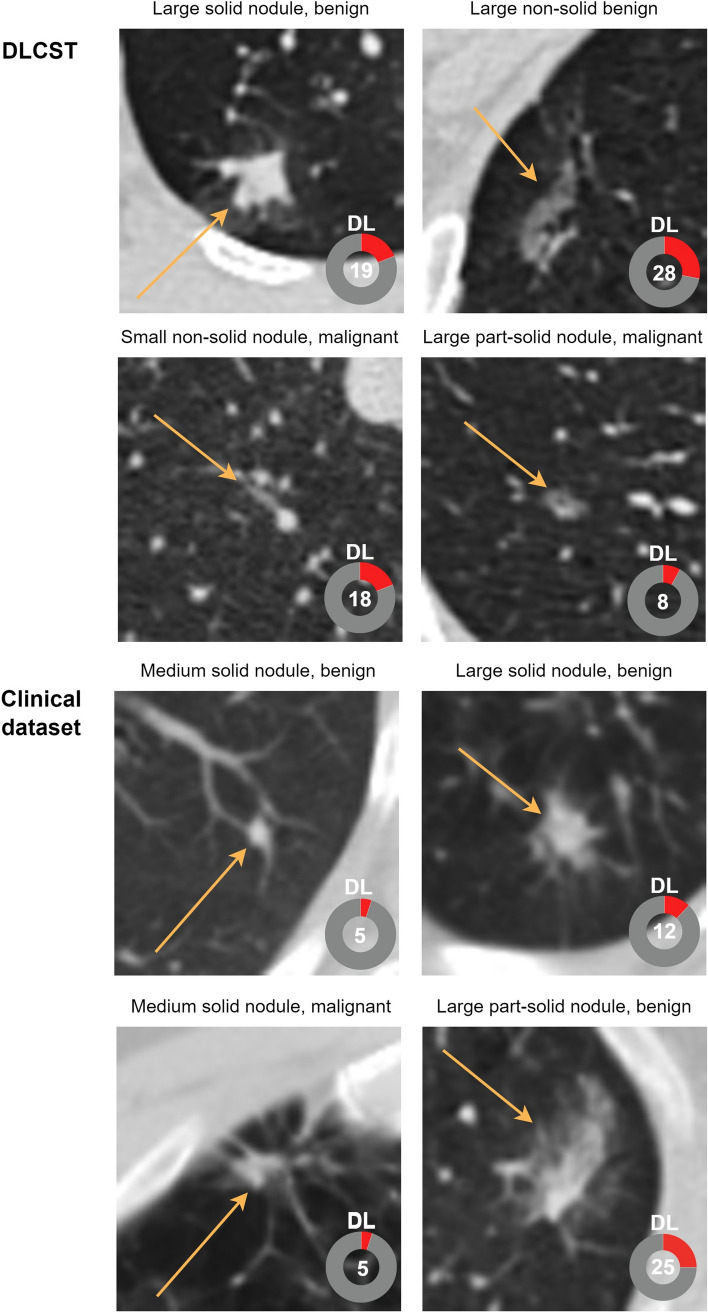
Examples of uncertain cases from the Danish Lung Cancer Screening Trial (DLCST) dataset and the Clinical dataset. Numbers in the bottom right corner of each image indicate the predicted DL malignancy risk, with an extent of color filling in the rings that is proportional to the malignancy risk. A malignancy risk of 0 represents the lowest risk, and 1 represents the highest risk. Arrows indicate the nodule location. DL: Deep Learning Malignancy Risk Estimation. Small: < 6 mm, medium: ≥ 6 to < 8 mm and large: ≥ 8 mm

**Fig. 6:**
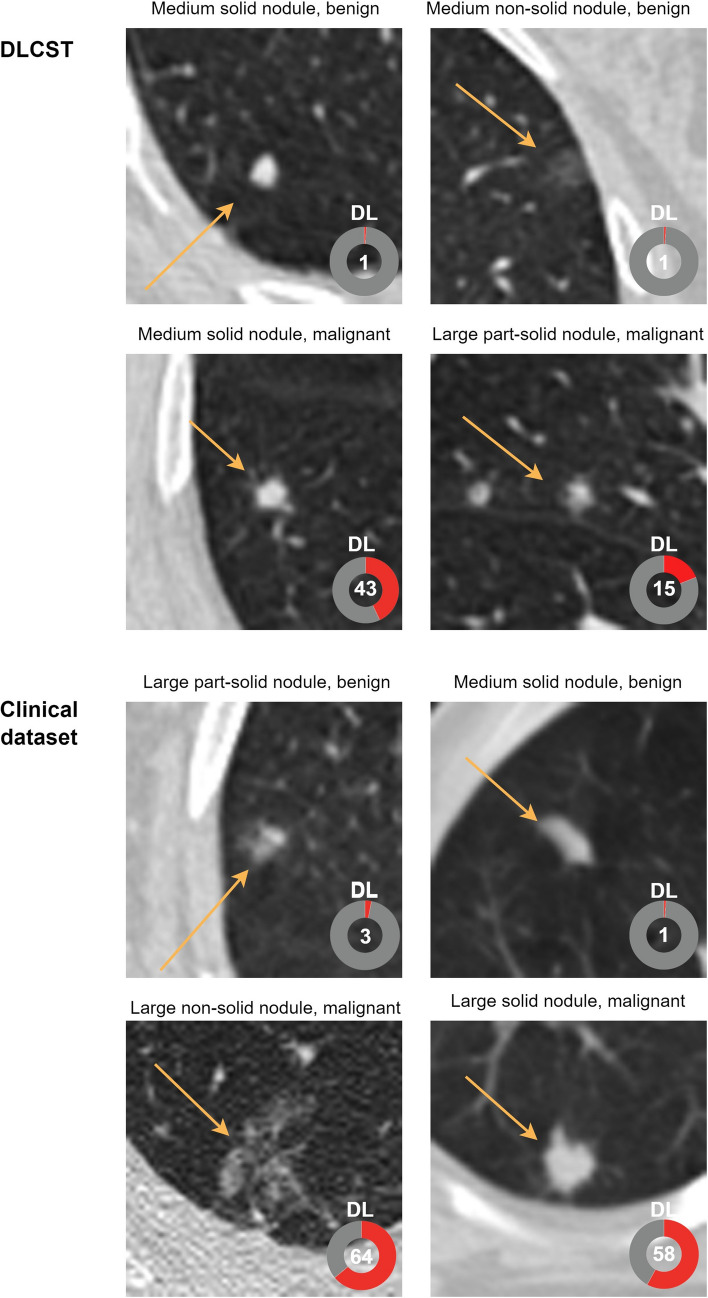
Examples of certain cases from the Danish Lung Cancer Screening Trial (DLCST) dataset and the Clinical dataset. Numbers in the bottom right corner of each image indicate the predicted DL malignancy risk, with an extent of color filling in the rings that is proportional to the malignancy risk. A malignancy risk of 0 represents the lowest risk, and 1 represents the highest risk. Arrows indicate the nodule location. DL: Deep Learning Malignancy Risk Estimation. Small: < 6 mm, medium: ≥ 6 to < 8 mm, and large: ≥ 8 mm

The DL algorithm achieved an AUC of 0.88 (95% CI: 0.84, 0.91) on the full clinical dataset (Figure 4 and Table 2). By applying the DLCST 90th percentile threshold on the clinical dataset, the algorithm achieved an AUC of 0.90 (95% CI: 0.86, 0.94) for the certain group and an AUC of 0.62 (95% CI: 0.50, 0.73) for the uncertain group. Using the DLCST 95th percentile threshold on the clinical dataset, the algorithm achieved an AUC of 0.89 (95% CI: 0.86, 0.93) for the certain group and an AUC of 0.64 (95% CI: 0.49, 0.78) for the uncertain group. DeLong’s test showed a significant difference in the performance of the DL algorithm between the certain group and the uncertain group of the clinical dataset for the 90^th^ and 95th percentile (*p* <.001 and *p* = .004, respectively). No significant differences were found between the full dataset and the certain group for the 90th and 95th percentile (*p* = .342 and *p* = .566, respectively). At 95% specificity, the DL algorithm achieved a sensitivity of 32% on the full clinical dataset, while a sensitivity of 34% was achieved on a certain group using the 90th percentile threshold of DLCST. Figures 5 and 6 show examples of nodules where the DL algorithm is certain and uncertain about, together with the malignancy label, DL output, nodule size, and type.

### Subgroup analysis

Table 3 summarizes the characteristics of the certain and uncertain nodules in the DLCST and clinical dataset. In appendix 1 and 2, a complete overview of the number and percentages of nodules considered certain and uncertain per subgroup is given. Nodules were categorized into subgroups based on their size (small, medium, large) and type (solid, part-solid, and non-solid). Analysis of the mean nodule size (mm) shows that in DLCST, the uncertain group consisted of significantly larger benign nodules than the certain group (*p* < 0.001). In the clinical dataset, the uncertain group also consisted of significantly larger benign nodules than the certain group (*p* = 0.02) and significantly smaller malignant nodules than the certain group (*p* < 0.001).

**Table 3:** Patient and nodule characteristics of the certain and uncertain group in DLCST and clinical dataset for benign and malignant nodules

**DLCST**				
	**Benign**		**Malignant**	
	**Certain (754)**	**Uncertain (64)**	**Certain (40)**	**Uncertain (25)**
Mean age in years	57.9 ± 4.7	58.6 ± 4.8	61.4 ± 4.6	62.5 ± 5.0
Sex				
Male	422 (56)	24 (38)	26 (65)	12 (48)
Female	332 (44)	40 (62)	14 (35)	13 (52)
Nodule Type				
Solid	659 (88)	36 (56) †	30 (75)	15 (60)
Part-solid	18 (2)	9 (14) †	6 (15)	6 (24)
Non-solid	77 (10)	19 (30) †	4 (10)	4 (16)
Nodule Size (mm)				
Mean	6.0 ± 4.5	12.3 ± 8.0*	15.9 ± 14.0	12.5 ± 7.9
Median	5.0 [4.0, 7.0]	10.0 [7.4, 15.0]	13.5 [9.5, 19.3]	9.5 [8.0, 12.5]
Nodule Size				
Small (< 6 mm)	453 (60)	9 (14)*	3 (8)	1 (4)
Medium (≥ 6 to < 8 mm)	160 (21)	9 (14)*	4 (10)	4 (16)
Large (≥ 8 mm)	141 (19)	46 (72)*	33 (82)	20 (80)
**Clinical dataset**				
	**Benign**		**Malignant**	
	**Certain (114)**	**Uncertain (53)**	**Certain (159)**	**Uncertain (48)**
Mean age in years	63.6 ± 11.8	64.2 ± 10.5	64.8 ± 9.3	64.0 ± 7.9
Sex				
Male	74 (65)	37 (70)	93 (58)	29 (60)
Female	40 (35)	16 (30)	66 (42)	19 (40)
Nodule Type				
Solid	100 (88)	36 (68) †	147 (92)	40 (83)
Part-solid	3 (3)	5 (9) †	8 (5)	7 (15)
Non-solid	11 (9)	12 (23) †	4 (3)	1 (2)
Nodule Size (mm)				
Mean	8.4 ± 7.1	11.2 ± 6.9*	30.4 ± 17.9	20.3 ± 15.6*
Median	5.7 [4.3, 10.0]	9.1 [7.0, 12.4]	24.7 [16.7, 41.8]	16.1 [11.7, 22.9]
Nodule Size				
Small (< 6 mm)	61 (54)	8 (15)*	0 (0)	0 (0)
Medium (≥ 6 to < 8 mm)	16 (14)	13 (25)*	2 (1)	3 (6)
Large (≥ 8 mm)	37 (32)	32 (60)*	157 (99)	45 (94)

Note. Data are numbers of nodules, with percentages in parentheses, except where otherwise indicated. When median numbers are reported, brackets indicate the inter-quartile range from the 25th and 75th percentile. When mean data are reported, ± indicates standard deviation. *: significant difference between certain and uncertain groups (*p*<.05). †: significant difference between Solid and Subsolid in certain and uncertain groups (*p*<.05)

Specifically focusing on the size-based subgroups, the DL algorithm demonstrated increased uncertainty when dealing with medium and large benign nodules as opposed to small benign nodules for both datasets (*p* = < 0.001). It also demonstrated increased uncertainty about part-solid and non-solid benign nodules compared to solid benign nodules for both datasets (*p* = < 0.01). For small solid, part-solid, and non-solid nodules in DLCST, only 1.9%, 0%, and 4.8% were considered uncertain, respectively. For large solid, part-solid and non-solid in DLCST, 18.1%, 62.5%, and 48.7% were considered uncertain, respectively. In the clinical dataset, 11.6% of the small solid nodules were considered uncertain in comparison with 23.9% of the large solid nodules. The clinical dataset did not contain any small part-solid or non-solid nodules. However, 52.2% and 46.2% of the large part-solid and non-solid were considered uncertain, respectively.

## Discussion

In this retrospective study, we integrated an uncertainty estimation method into a previously developed DL algorithm for estimating pulmonary nodule malignancy risk. We assessed the algorithm’s performance across two uncertainty thresholds, which were determined on screen-detected nodules from the Danish Lung Cancer Screening Trial (DLCST) and externally validated on incidentally detected nodules in a clinical setting. Using the 90th percentile entropy-based uncertainty estimation, the algorithm achieved an AUC of 0.93 (95% CI: 0.88, 0.97) for the certain group and an AUC of 0.62 (95% CI: 0.49, 0.76) for the uncertain group in DLCST. Externally validating this uncertainty threshold on the clinical dataset, the algorithm achieved an AUC of 0.90 (95% CI: 0.86, 0.94) for the certain group and an AUC of 0.62 (95% CI: 0.50, 0.73) for the uncertain group. Further analysis shows that benign nodules in the uncertain group were significantly larger when compared to the certain group, while only in the clinical dataset did malignant nodules had a significantly smaller size. In addition, there was a higher percentage of subsolid nodules in the uncertain group compared to the certain group. These findings highlight nodule characteristics that contribute to DL algorithm uncertainty.

Our uncertainty method showcased a significant decline in AUC for the uncertain groups, underscoring the capacity to identify cases where the DL algorithm harbors doubt in its predictions. These findings align with prior research that ensemble-based DL algorithms provide noteworthy uncertainty assessments by quantifying the prediction variability through approaches such as entropy [11, 14, 21, 22]. However, there is a pivotal difference between these previous studies and ours. We extended beyond the typical uncertainty assessment by providing an examination of nodule characteristics in the uncertain subset. This distinctive approach equips us with the means to not only identify uncertainty but also understand the factors contributing to it as we have seen in our subgroup analysis.

Our subgroup analysis revealed that the uncertain group comprised a higher percentage of larger benign nodules, alongside subsolid nodules. Remarkably, the total proportion of nodules classified as uncertain was 27% in the clinical dataset, whereas this figure was 10% for the screening data from DLCST. This discrepancy shows the elevated levels of uncertainty observed when the DL algorithm encounters nodules with different characteristics acquired in a hospital setting with diverse CT scan protocols instead of a controlled environment such as a screening trial. This further highlights the pivotal role of uncertainty estimation and the importance of validating DL algorithms in target cohort data that were not included in the training phase.

Our work has limitations. The first limitation is that only two uncertainty thresholds were applied. These thresholds were arbitrarily chosen to make sure the largest part of the dataset was determined as certain. Hereby, we intended to mimic a real-world scenario in which referring a high percentage of cases to human readers would limit and counteract the use of an DL algorithm. Nevertheless, the optimal threshold depends on the healthcare setting in which the DL algorithm is applied. We recommend that the optimal uncertainty threshold is discussed in a multi-disciplinary approach to achieve the best clinical benefits, but this should be assessed further in future work. Additionally, a reader study could also evaluate the optimal threshold that balances and maximizes the performance of both the DL algorithm and radiologists. The second limitation is that lung cancer diagnosis in the clinical dataset was obtained through the National Cancer Registry, to which we had to link based on lobe location and incidence date to find the nodule corresponding to the reported cancer. This could have contributed to the selection of more obvious malignancies that are larger in diameter since only nodules with high confidence of representing lung cancer were included.

The quantification of algorithm uncertainty holds valuable potential implications for both clinical practice and model development, as this allows these ambiguous scenarios to be addressed in a more informed and strategic manner. The demonstrated value of uncertainty estimation could help in the clinical implementation of DL algorithms, as this currently remains limited [23, 24]. Especially with the growing detection rate of pulmonary nodules due to an increase in CT examinations [25] and growing interest in lung cancer screening [2, 5, 26], uncertainty estimation could further amplify the benefits DL algorithms have on the anticipated demands of human readers. However, future work should investigate the implications of incorporating such uncertainty estimation within the workflow and performance of human readers using a reader study. Furthermore, additional external validation using newer multicenter data and evaluation among various algorithms is essential to solidify the applicability of this uncertainty estimation. With a similar approach as Alves et al (*Radiology*, 2023) [16], future research should extend this uncertainty estimation across multiple algorithms, imaging modalities, and cancer types.

In conclusion, we successfully integrated an uncertainty estimation method into a previously developed DL algorithm for nodule malignancy risk estimation and demonstrated that we can identify uncertain cases for which a notable decline in algorithm performance is observed. The nodule characteristics observed in the uncertain group emphasize the prevalence of larger benign nodules and sub-solid nodules, highlighting various factors that contribute to algorithm uncertainty. Integrating uncertainty estimation can be a promising method of mitigating errors, offering clinicians a vital tool to discern cases where expert consultation is needed. In addition, the characteristics of the uncertain cases provide valuable feedback for algorithm developers, highlighting areas where the training data for the algorithm can be extended to improve algorithm performance.

## Supplementary Information

Below is the link to the electronic supplementary material.

Supplementary file1 (PDF 248 KB)

## Abbreviations

AUC: area under the receiver operating characteristics curve
DL: Deep Learning
DLCST: Danish Lung Cancer Screening Trial
IKNL: Netherlands Comprehensive Cancer Organization
NLST: National Lung Screening Trial

## Declarations

### Guarantor

The scientific guarantor of this publication is Colin Jacobs.

### Statistics and biometry

No complex statistical methods were necessary for this paper.

### Study subjects or cohorts overlap

DLCST nodule cohorts have been previously described in Venkadesh et al. *Radiology*, 2020 (https://doi.org/10.1148/radiol.2021204433) and a subset of nodules from the clinical dataset are previously described in Chung et al. Thorax, 2018 (https://doi.org/10.1136/thoraxjnl-2017-211372).

### Methodology

- Retrospective
- Experimental
- Multicenter study
