# Supplementary material for: Enhancing a deep learning model for pulmonary nodule malignancy risk estimation in chest CT with uncertainty estimation
Source: Eur Radiol. 2024 Mar 27;34(10):6639–51. doi: 10.1007/s00330-024-10714-7 (PMC11399205; doi:10.1007/s00330-024-10714-7)

Enhancing a deep learning model for pulmonary nodule malignancy risk estimation in chest CT with uncertainty estimation.

ELECTRONIC SUPPLEMENTARY MATERIAL

Appendix 1: Number and percentages of nodules per subgroup for DLCST using the 90th percentile Entropy cut-off value.

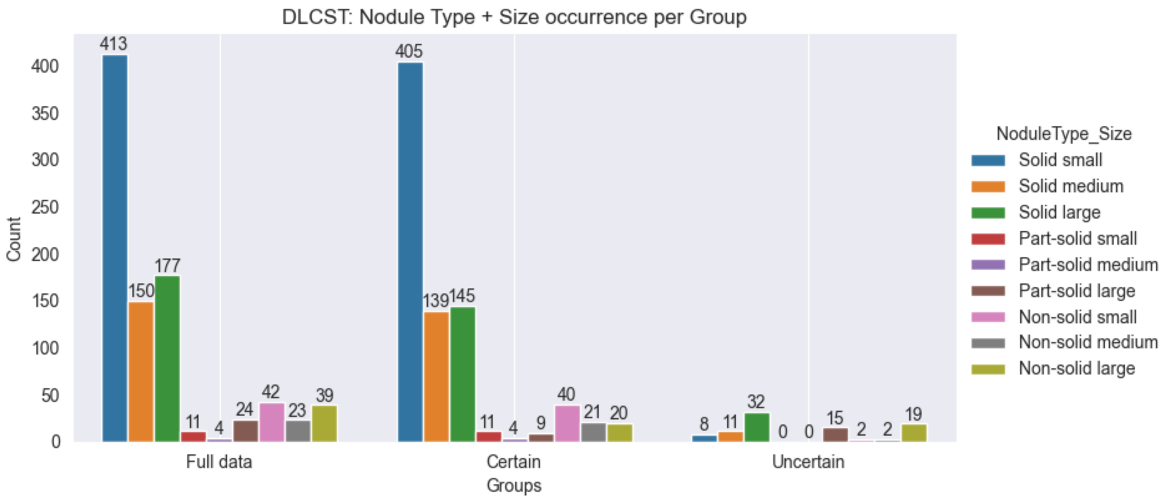

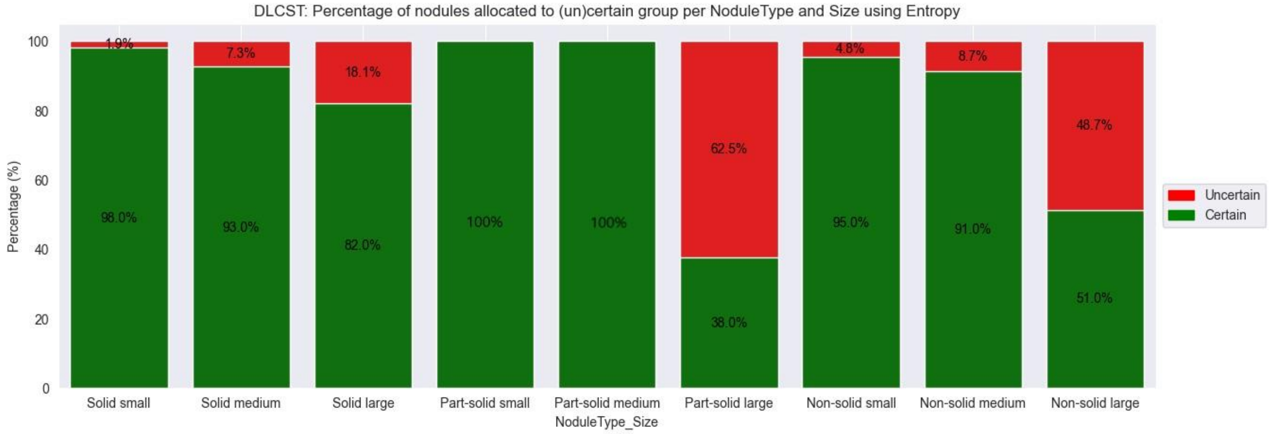

Appendix 2: Number and percentages of nodules per subgroup for the clinical dataset using the 90th percentile Entropy cut-off values.

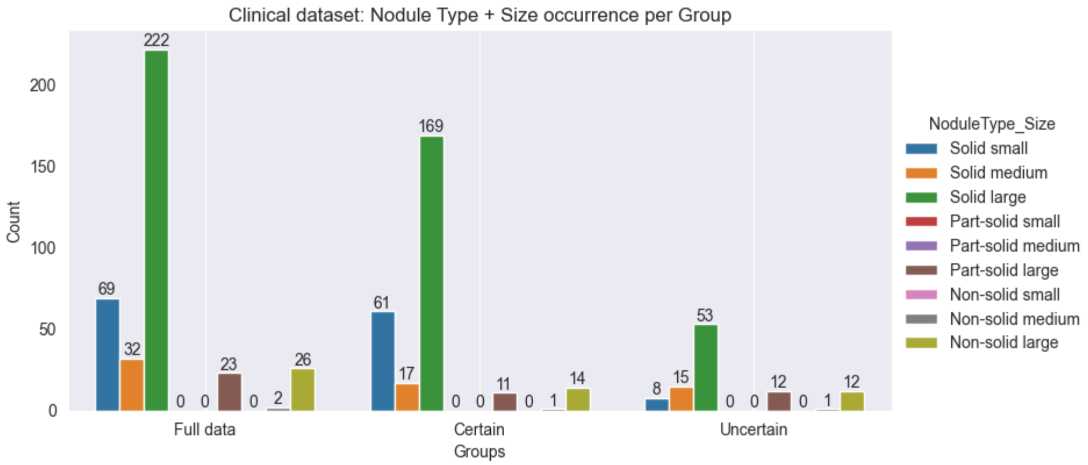

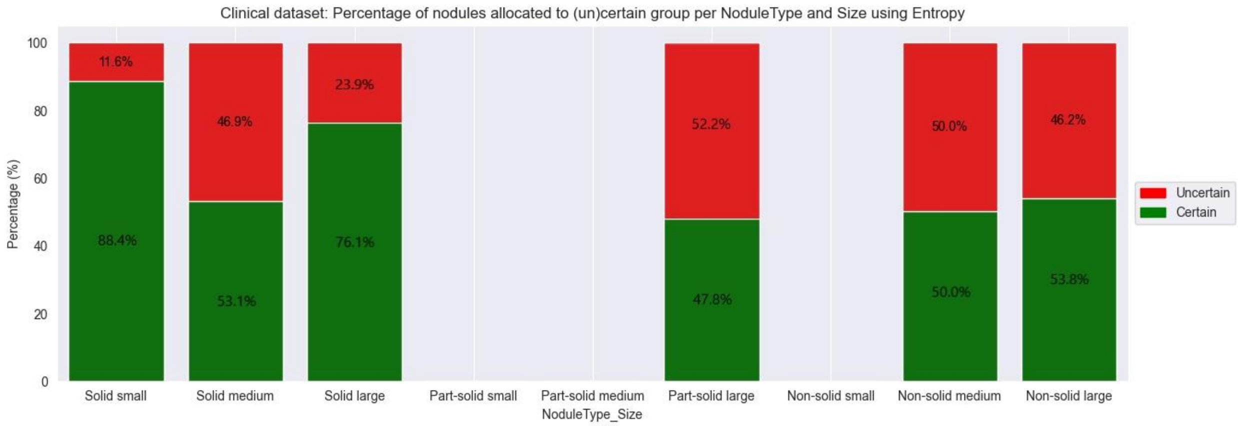

Supplement: Supplementary file 1 — Supplementary file1 (PDF 248 KB) [file 330_2024_10714_MOESM1_ESM.pdf]
